# Fiftieth anniversary of fiber optic–based fluorometry of brain mitochondrial NADH redox state monitored *in vivo*

**DOI:** 10.1117/1.JBO.30.S2.S23902

**Published:** 2025-02-19

**Authors:** Avraham Mayevsky

**Affiliations:** Bar-Ilan University, Leslie and Susan Gonda Multidisciplinary Brain Research Center, The Mina & Everard Goodman Faculty of Life Sciences, Ramat-Gan, Israel

**Keywords:** brain energy metabolism, mitochondrial function *in vivo*, laser Doppler flowmetry, brain physiological mapping, NADH fluorescence

## Abstract

**Significance:**

It is well known and accepted that the normal mitochondrial function in all cells in any organism is critical for the maintenance of cellular homeostasis. The development of *in vivo* technology to monitor mitochondrial function using nicotine–amide adenine dinucleotide (NADH) fluorescence started in the early 1950s. Until the early 1970s, the technology used for the light transfer between the light source and the monitored tissue as well as the detection system was very rigid and complicated. Monitoring of mitochondrial NADH redox states *in vivo* using the fluorescence approach could use a few techniques to transmit the light between the fluorometer and the monitored tissue.

**Aim:**

I describe the introduction of optical fibers as a tool to illuminate the monitored tissue as well as the light emitted from the tissue. I also present the advantages of using optical fibers.

**Approach:**

I describe in detail the introduction of ultraviolet (UV) transmitting optical fibers into the NADH monitoring system using various experimental protocols. The contact between the fiber optic probe and the monitored brain tissue was done by a special cannula cemented to the skull after removing a disk of bone in the parietal bone of the skull. In the same brain cannula, stainless steel electrodes, for electrocortical activity monitoring, were embedded in the wall of the light guide holder. The light guide holder was cemented to the skull by dental acrylic cement.

**Results:**

Using the fiber optic probe to monitor NADH fluorescence together with microcirculatory blood flow measured by laser Doppler flowmeter provided the new very unique types of results not published before.

**Conclusions:**

The introduction of UV-transmitting optical fibers, 50 years ago, to monitor tissue mitochondrial redox state opened up a new era in understanding the energy metabolism of tissues under *in vivo* conditions and in real time.

## 1 Introduction

### 1.1 Collaboration of AM with BC

My first meeting with Prof. Britton Chance was in Israel during his attendance at the Biophysical Society Meeting in 1971. He came to visit my advisor toward my PhD thesis, Prof David Samuel at the Isotope Department of the Weizmann Institute of Science, Israel. He saw the brain *in vivo* monitoring system for the evaluation of radioactive phosphate and immediately offered me a position in his group as a postdoctoral fellow at the Johnson Research Foundation in Philadelphia.

**Fig. 1:**
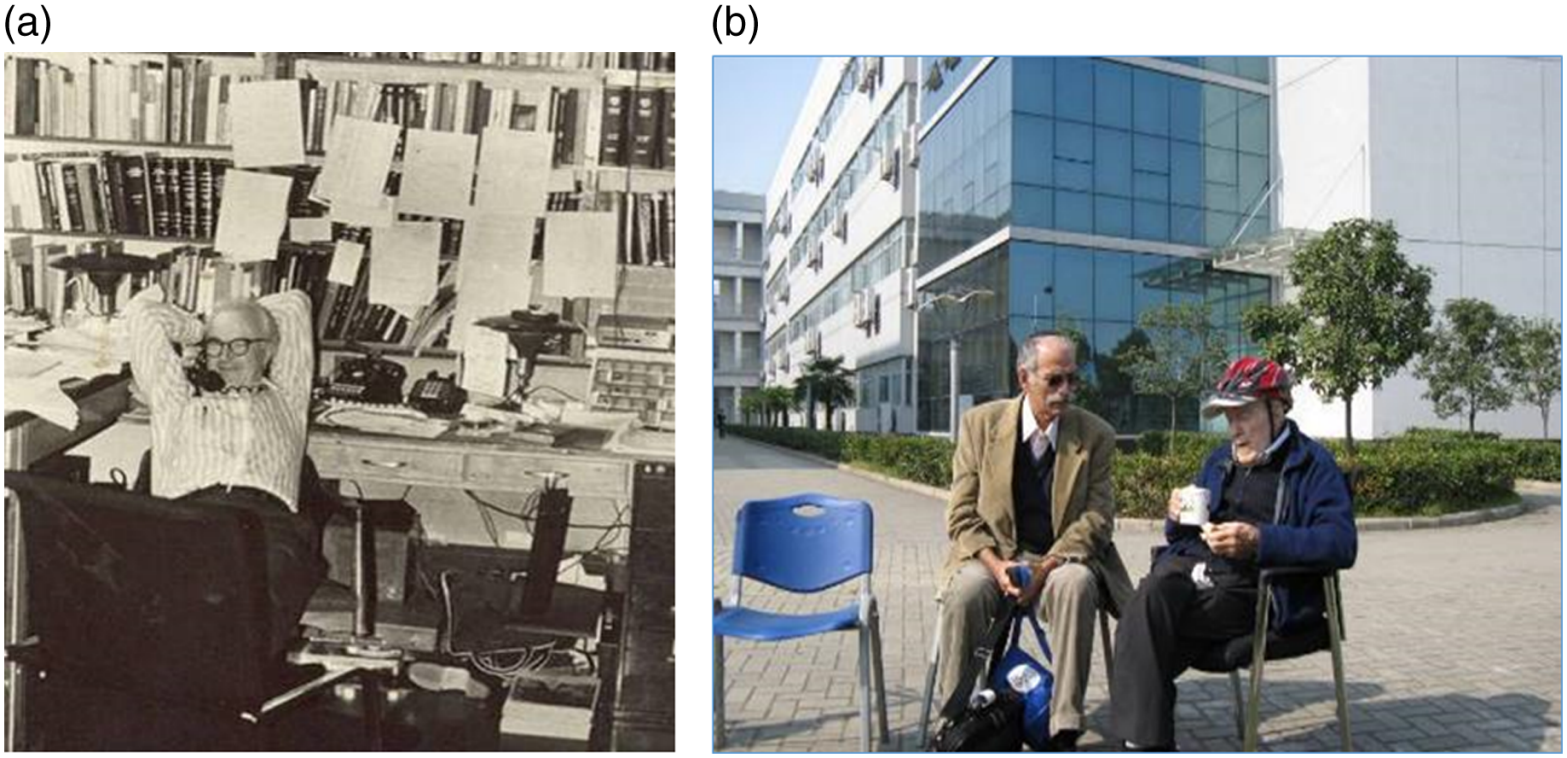
(a) Prof. Chance in his office at the University of Pennsylvania, Philadelphia (1973). (b) My (A. Mayevsky) meeting with Prof. Chance in Wuhan, China (2008).

After my graduation at the Weizmann Institute (Rehovot, Israel) in October 1972, I together with my family (wife and three children) moved to Philadelphia and stayed there for 2 years. I met Prof. Chance every day in his office/lab, as shown in Fig. 1(a).

After 2 years of post-doctoral activity, I went back to Bar Ilan University in Israel, but our collaboration continued, and my next visit was for a year from 1980 to 1981. Every year between 1974 and 1988, I visited the Johnson Research Foundation for an average period of 1 month. Later on, we spent 2 years in Philadelphia during which our first attempt to monitor Neurosurgical patients came through.

During my collaboration with Prof. Chance, we published 33 papers together, in addition to more than 100 papers that I published with other collaborators on nicotine–amide adenine dinucleotide (NADH) monitoring. Our meeting in 2007 to 2008 in China, as shown in Fig. 1(b), was sort of closing a life cycle, which started in Philadelphia in 1972 and ended in the famous Britton Chance Center for Biomedical Photonics, Wuhan National Laboratory for Optoelectronics (WNLO), Huazhong University of Science and Technology (HUST), Wuhan, China.

**Fig. 2:**
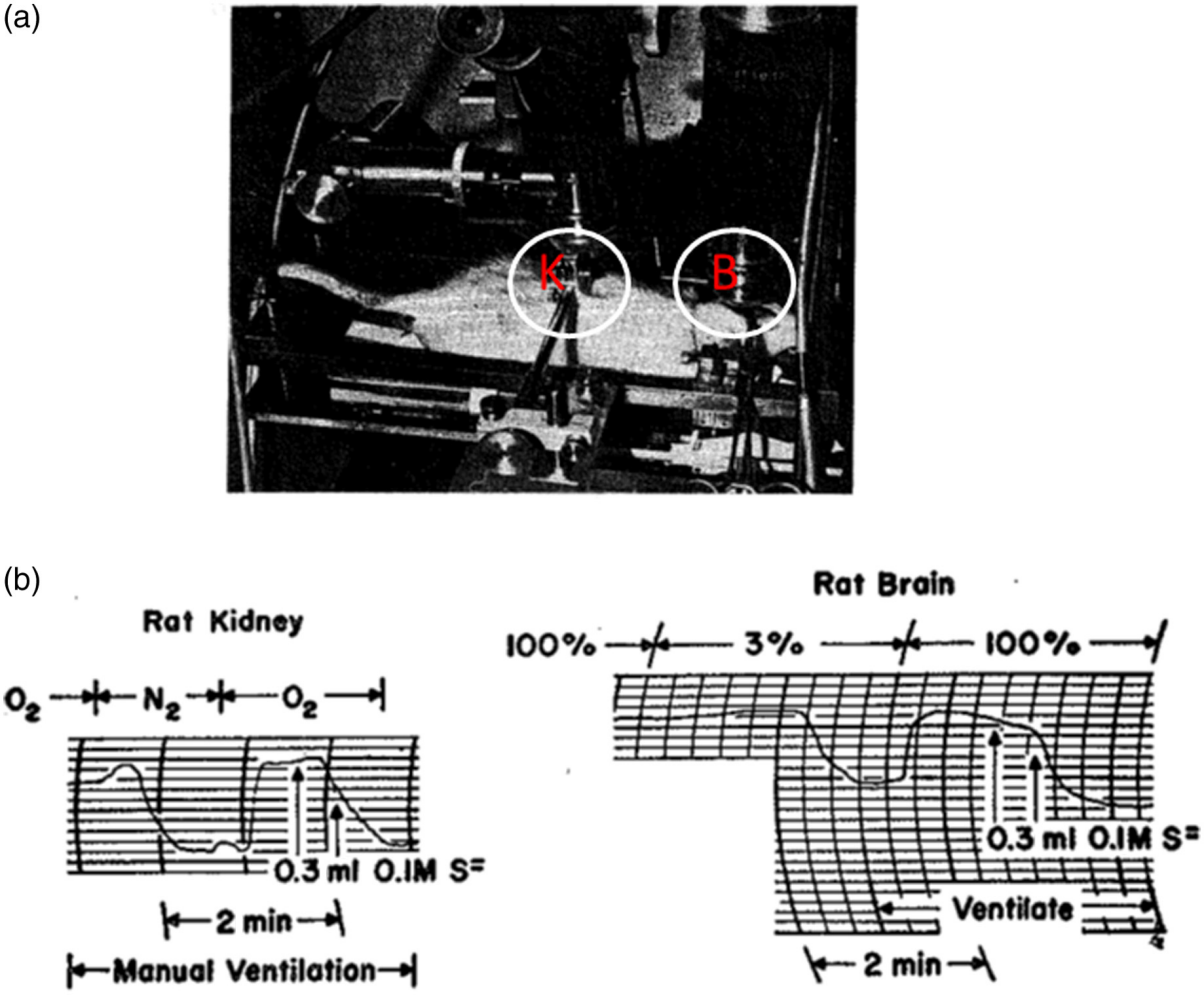
(a) Experimental arrangement for simultaneous microfluorometry of brain and kidney cortex in the rat. Two microfluorometers are focused on the exposed surfaces of the brain and kidney. By means of a tracheal cannula, the oxidation-reduction level of the intracellular pyridine nucleotide can be altered, and the corresponding fluorescence changes can be recorded by the two microfluorometers. (b) Microfluorometric recording of fluorescence increases caused by oxygen-nitrogen transition and by sulfide infusion into the vena cava, for the kidney (left) and brain cortex (right) of a urethane-anesthetized rat. The inspired gas was changed from oxygen to nitrogen for the kidney and from oxygen concentrations of 100% to concentrations of 3% for the brain. The time scale proceeds from left to right, and an increase in fluorescence is indicated as a downward deflection. In both experiments, the oxygen–nitrogen–oxygen transition is followed by a slow infusion of a solution of 0.1 M sulfide. The records indicate that the increase in fluorescence caused by sulfide inhibition of cytochrome oxidase is about the same as, or greater than, that observed in the oxygen–nitrogen transition, where hemoglobin is deoxygenated as well. The sensitivity in recordings on the brain is 2.5 times that in recordings on the kidney. ©Reprinted with permission from AAAS.^1^

Before describing my personal anecdotes with Prof. Chance, I would like to summarize his contribution to the field of bioenergetics by monitoring mitochondrial signals using light. He was the first to develop in detail an optical technique for the monitoring of mitochondrial signals, namely, NADH and Flavoproteins, in intact tissues and later on under *in vivo* conditions [Figs. 2(a) and 2(b)]. Prof. Chance started his activities in this field after the discoveries of Warburg and Keilin before 1950. He was the leader of modern biophotonics regarding the theoretical, experimental, and clinical application of optical monitoring of mitochondrial signals. During the first decade (1951 to 1962), he investigated the isolated mitochondria via tissues *in vitro* and finally under *in vivo* conditions. It is impossible to imagine the development of this field of bioenergetics without the foundations put together by Prof. Chance. In the 1970s, he started to apply optical technology to clinical applications.

I would like to mention a few events and episodes that represent my unique personal ties with Prof. Chance who was my second father (scientific).

A. In October 1972, we arrived in Philadelphia together with three children after an 18 h flight, and the Chance family hosted my family in their home for more than a week. This impressive welcome was very important in the establishment of my collaboration mode with Prof. Chance for more than 35 years. During this week, we had an opportunity to be in daily touch with the Chance family. Before dinner, Prof Chance (or one of the children) played the piano, which led to a very special atmosphere.
B. Prof. Chance was a very demanding scientist from himself as well as from his collaborators. One day when I was looking for the nitrogen cylinder at 7 pm during my experiments, Brit went with the cart to his lab and brought it to my lab. This kind of behavior stimulates our activities and fruitful collaboration. One day, he said, “I am waiting for your results for more than 10 years and I am pleased that you are running your studies intensively.”
C. After starting the routine experiments, we had a very stimulating meeting almost every evening. During this short session, Brit analyzed the results of the day, and we decided about the next day’s study.
D. In parallel to his demands, Brit took care of my scientific advancements and provided all my needs in the laboratory, and 3 months after my beginning, he took me to a meeting (head injury) at the NIH to present my preliminary results. Our first paper was published in mid-1973 after the ISOTT meeting in South Carolina.
E. Since day one of my collaboration with Brit, he emphasized the need for translation of the developed technology into clinical protocols and usage. This approach affected my efforts in this respect, and a few papers were published on the clinical monitoring of patients during neurosurgical procedures as well as in critical care medicine.

## 2 Methods

### 2.1 First Fiber Optic–Based Time-Sharing Fluorometer/Reflectometer

**Fig. 3:**
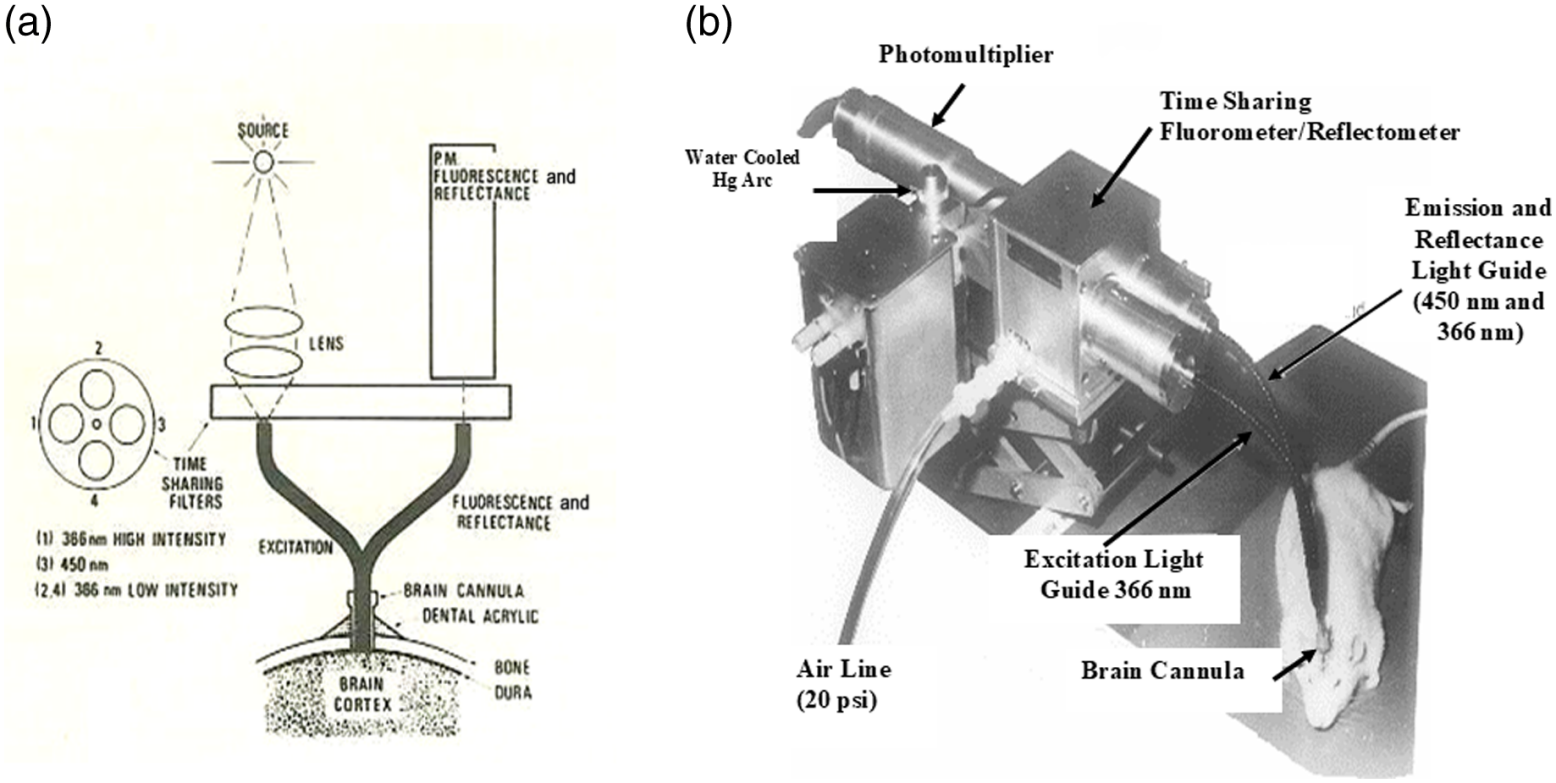
Structure of the first fiber optic–based fluorometer. In this model, the time-sharing principle was adopted and presented in panel (a). In panel (b), the fiber optic probe is connected to the brain of a small animal. ©Reprinted with kind permission of Springer Science & Business Media.^2^

**Fig. 4:**
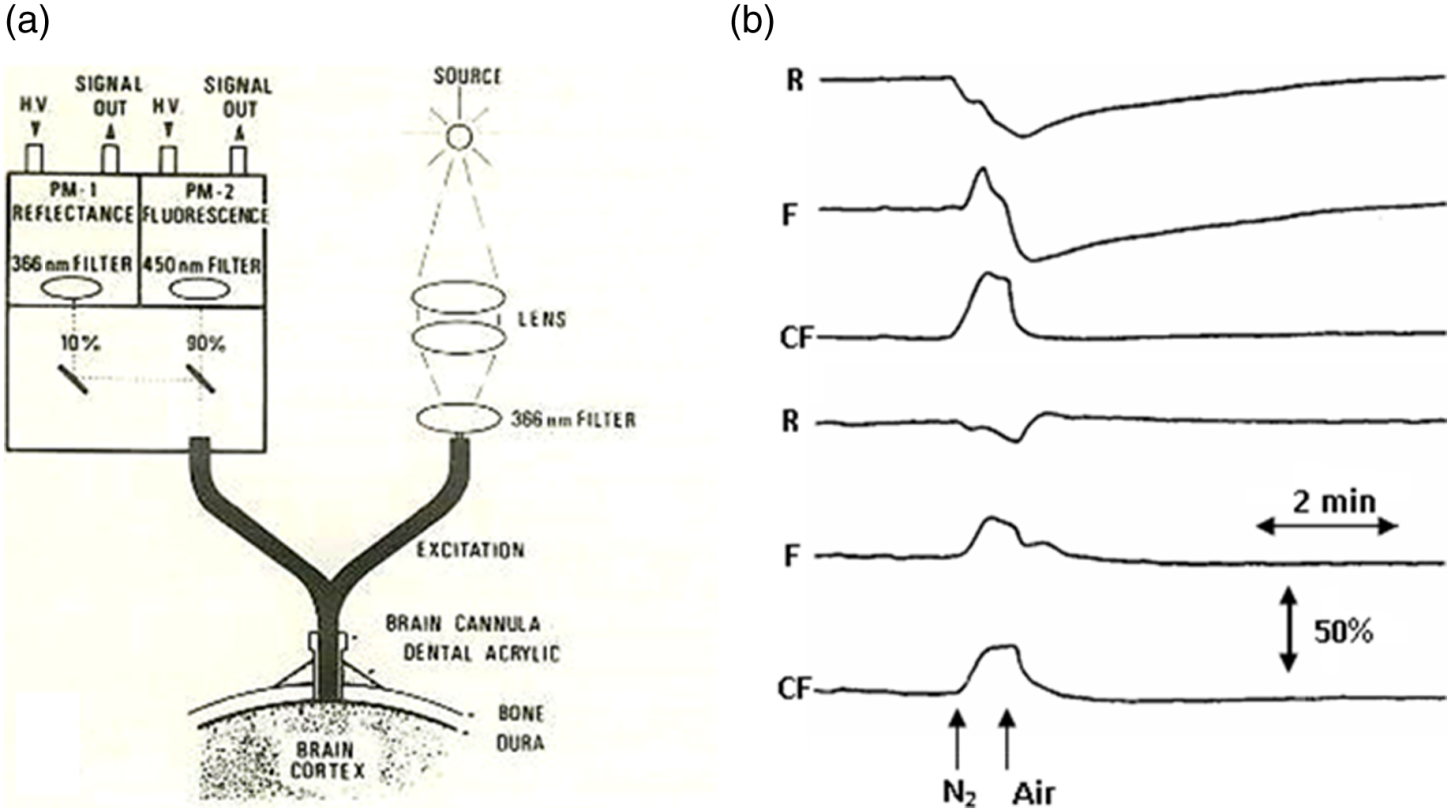
Standard DC fluorometer/reflectometer is shown in panel (a). The effect of the monitored tissue volume (the diameter of the fiber optic probe) was tested under anoxia as shown in panel (b) upper three traces (2-mm diameter) and (b) lower three traces (1-mm diameter). ©Reprinted with permission from Elsevier.^5^

Soon after my arrival in Philadelphia in late 1972, the first fiber optic–based fluorometer/reflectometer was constructed in the workshop at the Johnson Foundation unit and used in studying the rat’s brain *in vivo*, as seen in Fig. 3. To enable the monitoring of NADH fluorescence in unanesthetized animals or other *in vivo* preparations, a flexible means was needed to connect the fluorometer with the tested organ, for example, the brain. This was achieved in 1972 when ultraviolet (UV) transmitting quartz fibers became available (Schott Jena Glass, Germany). We have used the light-guide-based fluorometer for *in vivo* monitoring of the brain^3^^,^^4^ subjected to anoxia or cortical spreading depression (CSD). The historical development of light guide–based fluorometry reflectometry is shown in Fig. 3, the original device on the time-sharing principle [Fig. 3(a)], where four filters were placed in front of a two-arm light guide. Filters 1 and 3 enabled the measurement of NADH fluorescence, whereas filters 2 and 4 were used to measure tissue reflectance at the excitation wavelength. The reflectance trace was used to correct the NADH signal for hemodynamic artifacts and to indicate changes in the blood volume of the sampled tissue. In this original system, only one photomultiplier tube was used for the detection of the two signals. Figure 3(b) presents one of the first *in vivo* brain monitoring time-sharing setups, connected to the brain of an anesthetized rat.^2^ To simplify the monitoring system, the time-sharing approach (AC mode) was replaced by splitting the light emitted from the tissue into two unequal fractions for the measurement of fluorescence and reflectance signals. This model, named the DC-type fluorometer, had originally a three-way light guide, which was later replaced by a two-arm light guide probe [Fig. 4(a)]. In all three configurations, the reflectance signal was used for the correction of the fluorescence signal. The model shown in Fig. 3(a) was used to study the brain^2^^,^^4^^,^^6^^–^^9^ and kidney.^10^ The model shown in Fig. 4(a) was used to monitor the heart,^11^ brain,^12^^–^^14^ and kidney.^15^^,^^16^

Our group developed and used the model shown in Fig. 4(a) in the late 1970s. This model is still being used in our laboratory to monitor the brain,^17^^–^^20^ heart,^21^^–^^23^ liver,^24^ and kidney,^25^^–^^28^ as also in multi-site or multi-organ monitoring.^17^^,^^29^^–^^33^ The responses to anoxia using different diameters of the fiber optic bundle are shown in Fig. 4(b).^5^

## 3 Results

### 3.1 From Single Parameter to Multiparameter Monitoring Approach

**Fig. 5:**
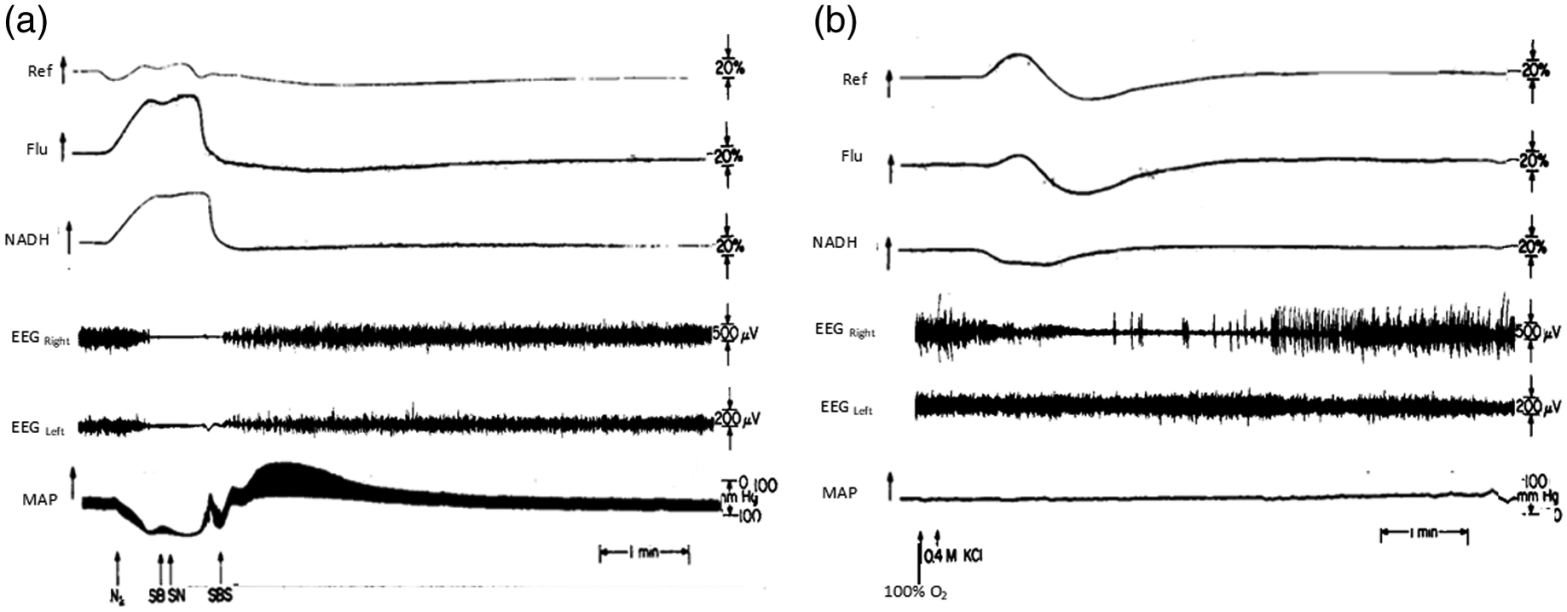
(a) Typical response of the rat brain and the systemic blood pressure (MAP) to a nitrogen cycle. Ref, reflection; Flu, fluorescence; MAP, mean arterial pressure; SB, stop breathing; SN, stop nitrogen; SBS, start breathing spontaneously. (b) The response of the brain to the application of KCl (0.4 M) on the dura surface under hyperoxia (100% $O_{2}$) to induce CSD.^34^

To correlate the changes in mitochondrial NADH redox state with another brain physiological activity, the measurement of brain electrical activity (electro cortical activity—EEG) was added to the monitoring system, as shown in Fig. 5.

**Fig. 6:**
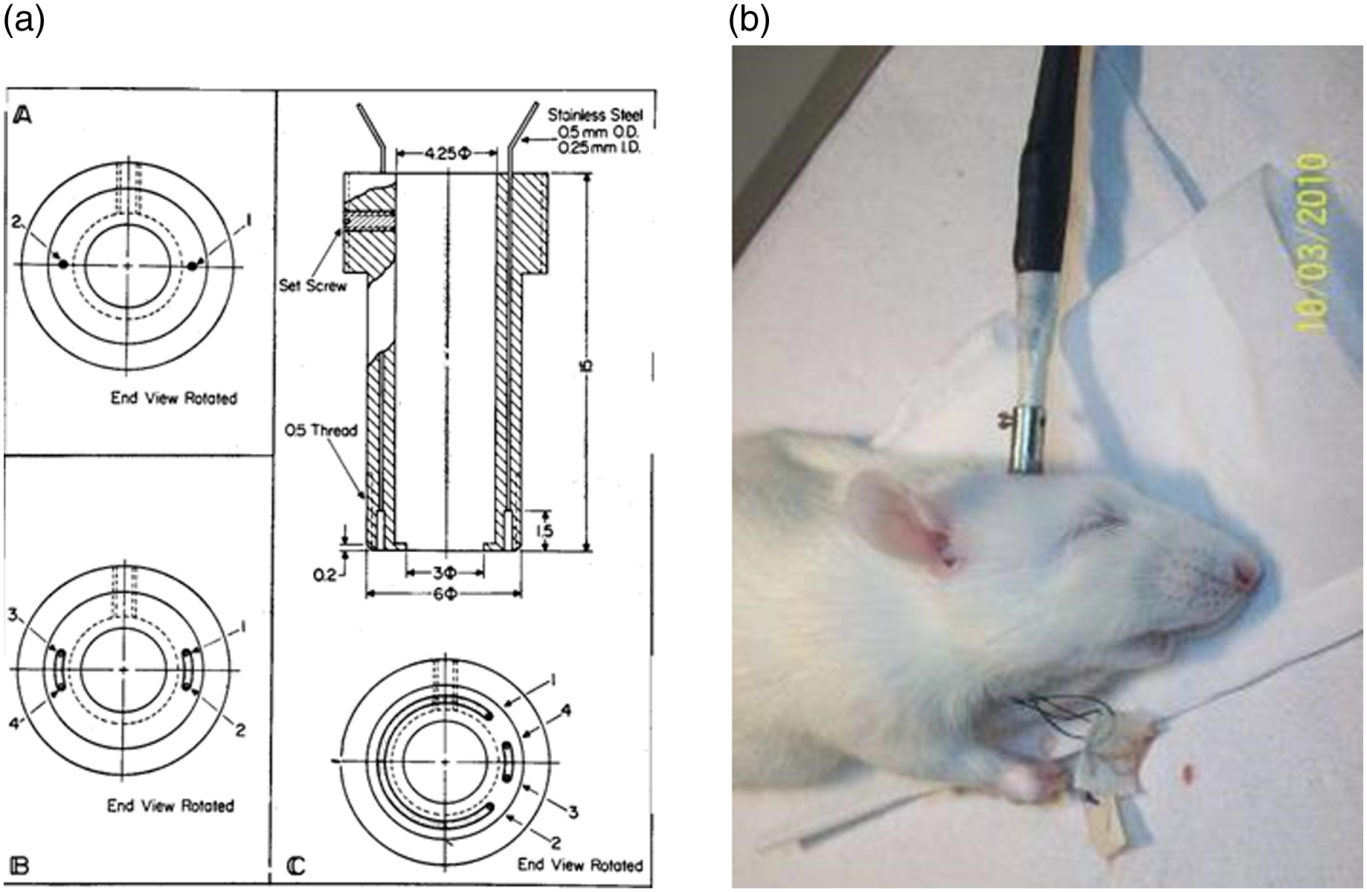
(a) Construction of the light guide holders (cannulas) for measurement of NADH fluorescence from the surface of the brain of an awake rat in experiments in which topical application of drugs is needed. (aA) The EEG is measured by the two electrodes 1 and 2. (aB) Two sets of stainless steel tubes 1 to 2 and 3 to 4 enable the measurement of the EEG and to wash solution of KCl to induce CSD. (aC) The four stainless steel tubes are located in different locations that enable to washing also pharmacological agents between tubes 1 and 2. ©Reprinted with permission from Elsevier.^6^ (b) After the insertion of the fiber optic probe into its holder, the animal is ready for monitoring. ©Reprinted with kind permission of Springer Science & Business Media.^35^

A special light guide holder (cannula) was designed, including a topical drug delivery system to the surface of the brain, as shown in Fig. 6.

The brain is operated, whereas the head is connected to a special head holder for the period of the operation (20 to 30 min) and then could be released, for the monitoring period, as shown in Fig. 6(b). The other monitored organs, i.e., muscle, kidney, or liver, have to be held by a micromanipulator during the monitoring period.

As mentioned before, the brain was the main organ monitored by other investigators as well as by our group. To be acquainted with the NADH monitoring device, it is recommended to start with brain monitoring and later on to move and use the selected organ. The reason for this is that the connection between the fiber optic probe and the monitored tissue must be constant during the monitoring session. In the brain, it is easy to achieve an optimal measurement by connecting a probe holder to the skull using acrylic cement, as shown in Fig. 6(b).

A midline incision is made in the skin, exposing the skull. Three holes were drilled in the skull, and appropriate small screws were inserted into the skull (less than 1 mm in depth). An appropriate hole (3 to 5 mm in diameter) was drilled in the right or left parietal bone for the fixation of a light guide holder in which the monitoring probe was later inserted. The light guide holder and the three screws were then fixated to the skull using dental acrylic cement. Ten minutes later, the head of the animal was released from the head holder, and the probe was inserted to a predetermined depth and fixed by a set screw [Fig. 6(aA)].

The structure of the standard DC fluorometer/reflectometer connected to the brain of a small animal was shown.^36^ Various types of light guide holders are presented.^6^ The thread outside the bottom of this cannula enabled screwing it into the skull and also gave a better connection between the cannula and the cement. The cannula shown in Fig. 6(aA) was used in experiments in which animals were exposed to nitrogen/oxygen breathing cycles or to hyperbaric pressure of oxygen. The electroencephalogram (EEG) was measured between the two electrodes; 1 to 2 cannula type AB was used in all experiments in which spreading depression was elicited by the application of potassium chloride (KCl) solution above the dura. The four small electrodes 1, 2, 3, and 4 were small stainless tubes for the KCl application. The third type, 6AC, has two compartments in the bottom, the small one for KCl applications 3 to 4 and the large compartment for 1 to 2, where chemicals such as Metrazol were applied, affecting a larger area of the brain. A fifth electrode was located 180 deg from electrodes 3 to 4, so the EEG was measured at the same time.

We have used a short anoxic episode (20 to 30 s) to test the intactness of the tissue. If the NADH response to anoxia was too small or very large compared with the average response, we stopped the experiment because it indicated that the brain was not in a good physiological condition.

### 3.2 Fiber Optic–Based Fluorometer and EEG

During the initial step, we developed the fiber-optic-based fluorometer/reflectometer for monitoring mitochondrial NADH in anesthetized or unanesthetized animals. Various types of fiber optic probes were developed during the years. To keep direct and constant contact between the brain and the probe, special design holders were developed over the years. The first fiber optic probe was described in 1973,^3^^,^^5^ and the other types were presented in our review paper.^5^^,^^37^ After the end of the operation, the probe was inserted into the holder that was cemented to the skull.

Figure 5 shows the typical two responses obtained in a rat experiment. As can be seen, we monitored two signals by the fluorometer, namely, the NADH fluorescence at 450 nm and the total backscattered light at the excitation wavelength (366 nm) called reflectance. In addition to NADH, we monitored EEG activity by placing two stainless steel electrodes on the brain surface. In these preliminary studies, the increase in the fluorescence and reflectance signals was in the down deflection compared with the upward direction used in the other records in the book.^38^

In Fig. 5(a), the complete elimination of oxygen led to a large increase in the NADH, and the EEG signal disappeared very fast. In this animal, the change in the reflectance was very small compared with most of the rats. As soon as the rat started to breathe air, the NADH level recovered very fast. In panel (b), the effect of brain activation induced by CSD is shown. In this response, the blood volume changes concomitantly with the mitochondrial NADH. The NADH redox state was shifted to a more oxidized state due to the increase in Adenosine triphosphate (ATP) demand. The CSD developed only in the stimulated hemisphere, as seen in the EEG signal, whereas the contralateral hemisphere served as a control. The same monitoring system was used in other studies.^39^

**Fig. 7:**
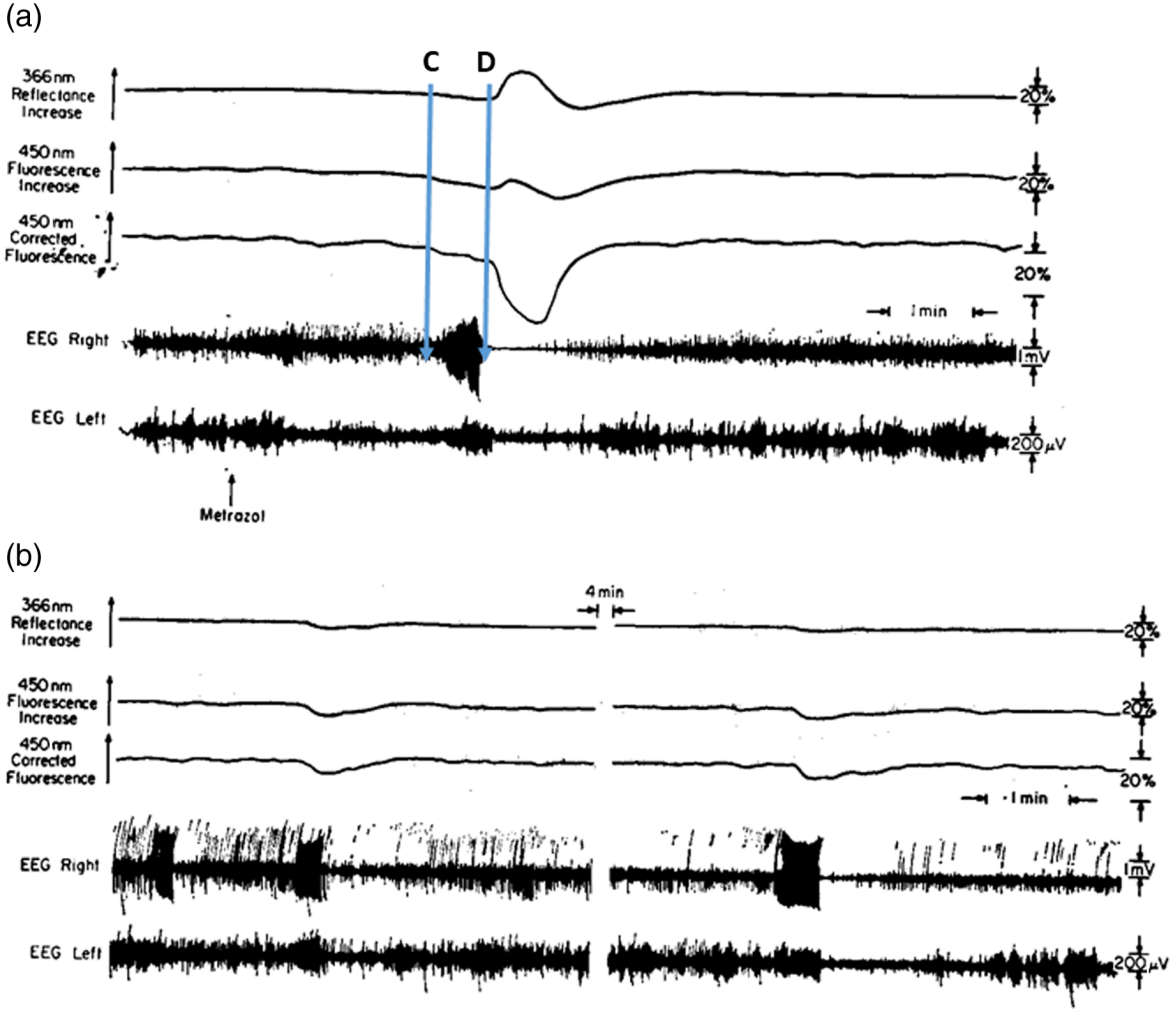
Effects of Metrazol on brain NADH fluorescence, 366-nm reflectance, and the EEG of the two hemispheres. Note that the amplitude of the EEG calibration in the right hemisphere is 1 mV. The typical response is shown in panel (a). Panel (b) shows another type of response to Metrazol applied 10 min before the record was taken.^6^

In 1975, we published our first paper on mitochondria NADH responses to epileptic activity measured in non-anesthetized rats.^6^ The Metrazol was applied epidurally to the surface of the rat brain, as shown in Fig. 7(a). The Metrazol (100 mg/ml) was applied epidurally using the cannula shown in Fig. 5. The typical response to Metrazol occurs 3 to 5 min after the administration of the drug, and the results are shown in panel (a). The Metrazol was applied to the right hemisphere, and the left one served as a control. After application of the Metrazol, an increase in electrical activity was recorded, and as a result, oxidation of NADH, which was in the range of 5% to 10% of the normoxic level. After a period of very intense activity, the EEG became isoelectric, whereas the NADH showed a very large oxidation cycle, which then recovered to the normoxic level. In some animals, a different response was recorded, and it is shown in panel (b). In these cases, the small oxidation under the Metrazol effect was recorded, but the recovery to the normoxic level showed no large oxidation cycle. The reflectance changes were very small during the effect of Metrazol.

### 3.3 Fiber Optic–Based Multiparametric Monitoring System

The multiparametric monitoring system was developed mainly to study the brain, but the same concept and technology were used in other organs such as the heart and kidney. This system includes a multiprobe assembly (MPA) that describes various sensors that are located on the surface of the brain. The aim of the MPA is to provide real-time data describing the relationship among hemodynamic, metabolic, ionic, and electrical activities in the cerebral cortex. Normal brain mitochondrial function is a precondition for the performance of all other brain functions. Therefore, a short introduction to brain energy metabolism is presented here. The aim of this part of the paper is to demonstrate the historical development of the technology used in monitoring the brain and other organ functions under various pathophysiological conditions. We are presenting the stages of the development and typical results obtained in our laboratory. It is important to note that, in our monitoring system, all the probes were placed on the surface of the brain and never penetrated the tissue itself. Most of the references cited in this section were published by our group.

#### 3.3.1 Brain energy metabolism

**Fig. 8:**
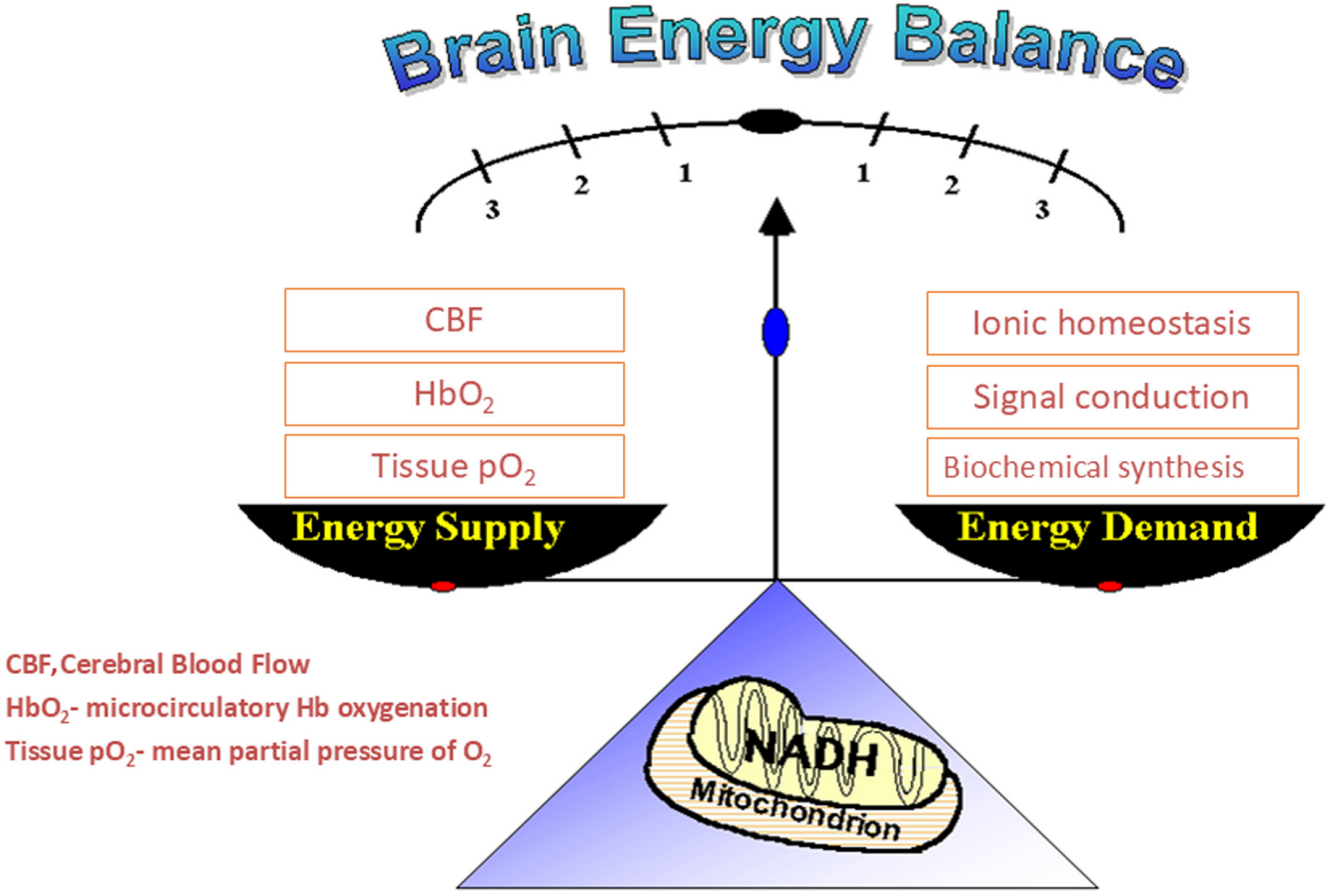
Schematic presentation of the brain energy balance concept linked to energy supply and demand. The mitochondrial NADH redox state represents the balance between oxygen supply and demand.^40^

The functional capacity of the brain is related to its ability to perform its work. It is possible to assess this ability through the knowledge of changes in the oxygen balance, i.e., the ratio between oxygen supply to oxygen demand in the brain. Healthy brain cells perform various types of activities, as presented on the right side of Fig. 8 (energy demand). The energy is derived through several complex enzyme systems, in which oxygen is the ultimate electron acceptor. The electron transfers down the respiratory chain result in the production of ATP. Concomitantly with the electron transport, the respiratory chain components switch between reduced and oxidized states, each of which has different spectroscopic properties. The formation of the pyrophosphate bonds depends on the sufficiency of sugar and oxygen functions, with inadequacy that can, ultimately, lead to death. As most of the energy consumed by tissues is dependent on the availability of oxygen, the terms “energy” and “oxygen” are used here synonymously. In a normal healthy brain, the ratio or balance between oxygen supply and oxygen demand is positive and reflects the brain cell’s functional capacity to do work. That is, the supply mechanism of blood flow and the oxygenated blood circulation is able to provide the spectroscopic properties of the respiratory chain components that are unique to their redox status and used as internal markers of the state of oxidative phosphorylation.

In excitable tissues, such as brain or muscle tissue, as well as in other cells, the activity of Na-K-ATPase is very sensitive to alterations in ionic homeostasis. An increase in extracellular potassium ion concentration, $K^{+}$, will stimulate pumping activity to bring the extracellular $K^{+}$ back to normal levels, i.e., 3-mM range. The activation of Na-K-ATPase will increase the hydrolysis of ATP, and thus, the mitochondria will phosphorylate the ADP molecules that are released. The accelerated activity of the mitochondria will be accompanied by a more oxidized state, more oxygen delivery to the cells, blood flow, and blood supply. This coupling between energy consumption and energy production is maintained as long as the $O_{2}$ supply is well regulated.

**Fig. 9:**
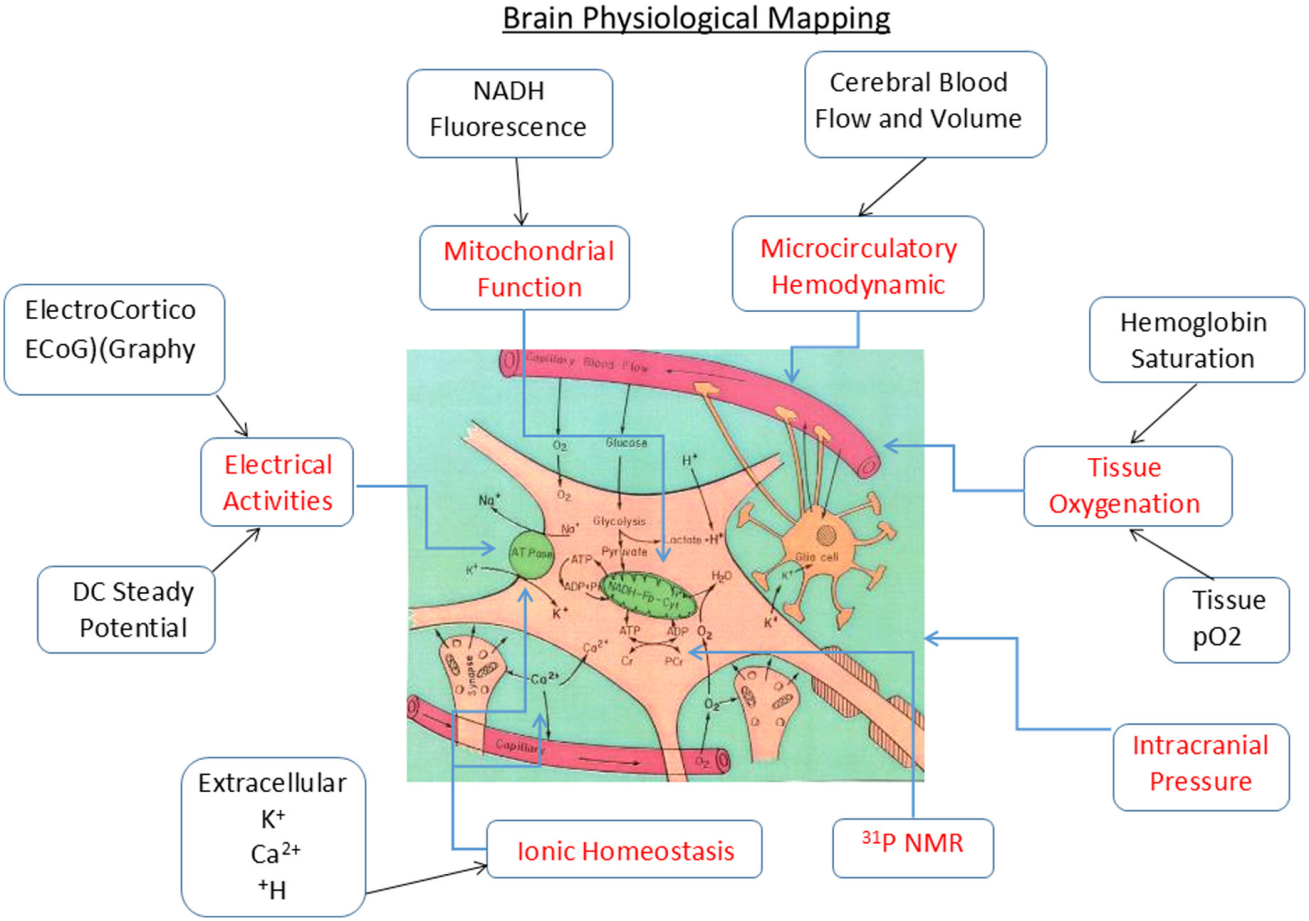
Schematic presentation of the concept of “brain physiological mapping.”^40^ All techniques that are presented were developed and used in our laboratory.

Under conditions where the oxygen supply or delivery is limited, e.g., after a stroke or heart attack, the energy supplier, i.e., the mitochondria, will not be able to produce the amount of ATP needed. As a result, energy-demanding processes will be restricted. The net effect of the imbalance between energy demand and supply will be manifested by a decrease in the tissue’s ability to do work. This can lead to the development of various pathological states. The left side of Fig. 8 presents the various parameters that could be monitored in addition to mitochondrial function as representative of oxygen supply. To evaluate brain oxygen balance, it is necessary to measure parameters that represent the oxygen supply and demand at the same time. The mitochondrial NADH redox state represents the supply as well as the balance. Therefore, it is necessary to measure more parameters in addition to mitochondrial NADH. As can be seen in Fig. 9, our approach aims to monitor, in real time, a small volume of the cerebral cortex containing all the tissue elements that are parts of a typical functioning brain.

#### 3.3.2 Brain physiological mapping

The term “brain physiological mapping” is used in the current paper to describe the relationship among the various monitored parameters in a small brain area and not the imaging of the whole brain. We are interested in the microenvironment of the brain containing neurons, glia, synapses, and microcirculatory elements (small arterioles and capillaries) in a small brain volume. The various parameters and the technology developed are presented in Fig. 9. During the development process, we pursued the goal of being minimally invasive in terms of penetration to the cortical tissue itself. It was obvious that the various probes could not monitor the same volume of tissue due to the size of each probe used. Therefore, we attempted to minimize the diameter of the various probes located in the MPA that had a 5- to 6-mm contact area with the cerebral cortex. In most of the perturbations used, such as global ischemia, anoxia, hypoxia, or hemorrhage, most of the areas in the cortex will respond in the same way. We have tested this concept by the development of the multi-site monitoring of NADH and other parameters, as was presented in chapter 6 of our published book.^38^ As can be seen in Table 1, the initial step in the development of the MPA was the establishment of the fiber-optic-based NADH monitoring system in 1972 when the first UV-transmitting optical fibers appeared. It was a continuation of the long-term usage of old devices for NADH monitoring *in vivo* where the animal was located in an optic-based rigid device. The connection of the brain to the fluorometer via optical fibers enabled us to monitor, for the first time, the brains of unanesthetized animals. The initial data on the use of this technology appeared in two papers.^3^^,^^34^

**Table 1:** Milestones in the development of brain multiparametric monitoring of NADH fluorescence and other physiological parameters *in vivo* by Mayevsky et al.

Protocol	Year	Discovery/activity	Author(s)
1	1973	The first fiber optic–based fluorometer reflectometer used in the brain of an unanesthetized animal. Monitoring of NADH and electro cortical activity (ECoG)	3 and 4
2	1974, 1977	Simultaneous monitoring of NADH *in vivo* together with extracellular $K^{+}$ (microelectrode and surface electrode) and ECoG	7 and 41
3	1980	Monitoring of brain NADH together with tissue ${\mathrm{pO}}_{2}$ and ECoG	42
4	1982	The first multiparametric assembly for NADH, extracellular $K^{+}$, $H^{+}$, DC steady potential, and ECoG	43
5	1983	Monitoring of NADH, ${\mathrm{pO}}_{2}$, extracellular K+, DC, and ECoG inside hyperbaric chamber	44
6	1990–1992	Simultaneous real-time monitoring of brain NADH, ${\mathrm{HbO}}_{2}$, ECoG, DC potential, extracellular $K^{+}$ and ${\mathrm{Ca}}^{+2}$	45 and 46
7	1995	Simultaneous monitoring of brain NADH, CBF, ECoG, DC potential, extracellular $K^{+}$, ${\mathrm{Ca}}^{+2}$, $H^{+}$	47
8	1996	Multiparametric monitoring of neurosurgical patients	48
9	1997	Monitoring of brain NADH, CBF, DC potential, extracellular $K^{+}$, ${\mathrm{Ca}}^{+2}$ together with high-energy phosphates by P31 NMR spectroscopy	49
10	2000	Monitoring of the mechanism of CSD propagation	50
11	2001	Multiparametric monitoring under ICP elevation	51
12	2003	Multiparametric monitoring of rats under traumatic brain injury	52

The list in Table 1 is organized in chronological order. All details of the technological aspects and animal preparation appear in the original relevant publications; therefore, a short description of technology relevant to each parameter will appear in the initial part of Sec. 2. In Sec. 3, typical responses to various types of perturbations will be presented together with the technology used in the specific study. Our approach was to develop a new upgraded version of the monitoring system and present initial preliminary results. The next step was to run a large well-designed study on a few groups of animals, and the data were quantitated and analyzed for its statistical significance.

**Fig. 10:**
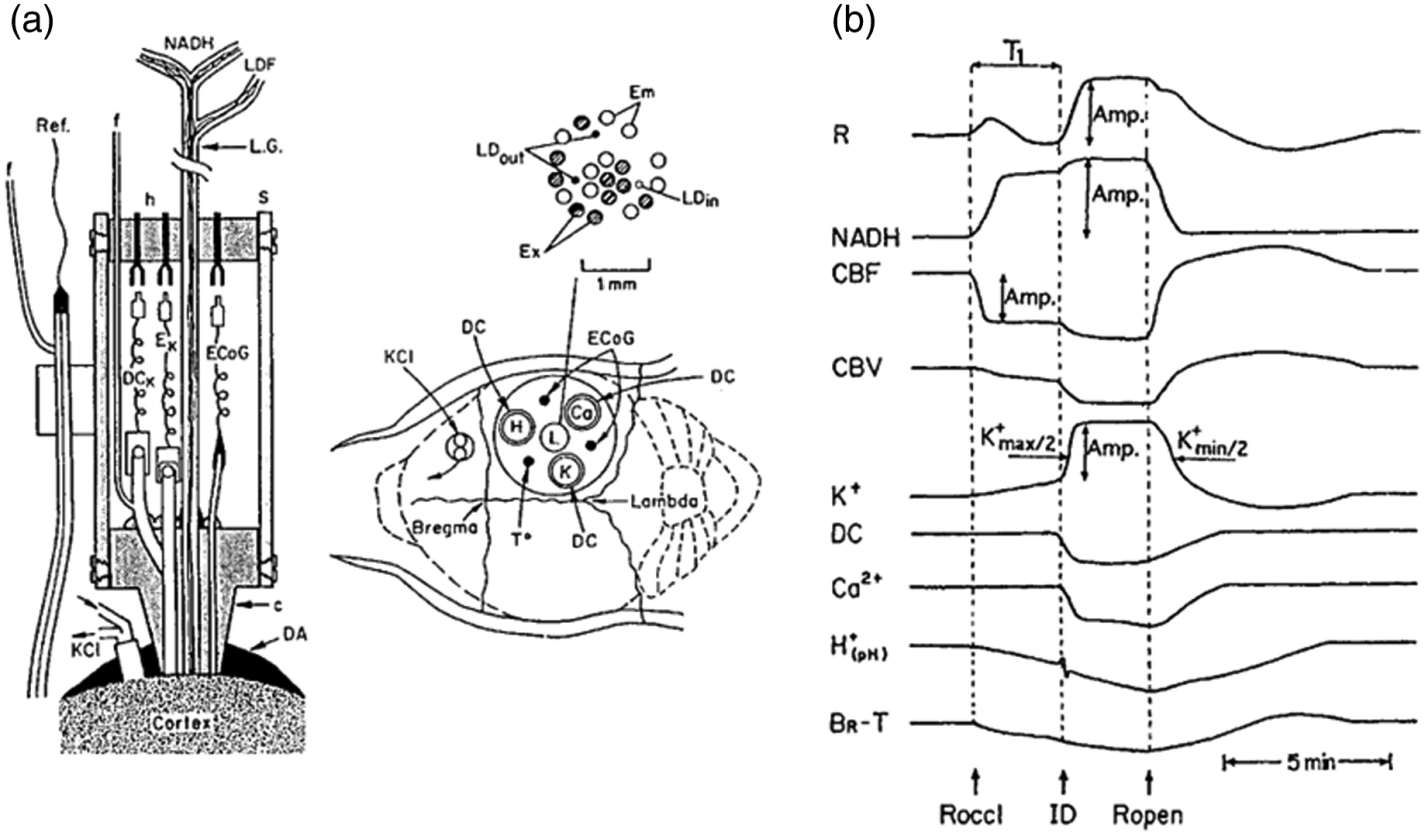
(a) Multiprobe assembly used to monitor hemodynamic, metabolic, ionic, and electrical activities from the brain. The left side shows a longitudinal section. In the right part, the location of the MPA and the skull is shown. c, Plexiglas probe holder; DA, dental acrylic cement; KCI, push–pull cannula for KCl; ECoG, electrocortical electrodes; K, Ca H, ion, specific electrodes; DC, area of DC steady potential monitoring; NADH, two arms of NADH monitoring light guide; h, connectors holder; s, aluminum sleeve; Ref, reference electrode; f, reeling tube of the reference or the other DC electrodes; To, thermistor for local temperature measurement; Ex, Em excitation and emission fibers for NADH monitoring; ${\mathrm{LD}}_{\text{in}}$ ${\mathrm{LD}}_{\text{out}}$, optical fibers for monitoring blood flow and volume; LDF, laser Doppler flowmeter fibers; L.G, combined light guide.^53^ (b) Schematic representation of the typical responses to ischemia in the Mongolian gerbil. R, reflectance; NADH, 450-nm corrected NADH; CBF, CBV, cerebral blood flow and volume; $K^{+}$, ${\mathrm{Ca}}^{2+}$ $H^{+}$, extracellular levels of ions; DC, steady potential; BR-T, brain temperature; $R_{\mathrm{occl}}$, $R_{\text{open}}$, occlusion and opening of the right carotid artery; ID, ischemic depolarization.^53^

To save space, we are presenting here only one set of typical results collected during the developmental stage (Fig. 10). In 2000, Mayevsky et al.^53^ published a review paper, in which real-time evaluation of drug tissue interaction *in vivo* was tested. A MPA shown in Fig. 10(a) was used.^53^ This system (described in detail in chapter 5 of my book)^38^ could be used in various animal models subjected to different perturbations and drug treatment.

In Fig. 10(b), the potential of the MPA in a gerbil brain ischemic model for compound testing is presented. To test the potential protection activity of various drugs, the gerbil brain was exposed to bilateral unilateral carotid artery occlusion.^30^^,^^54^ Figure 10(b) presents, in a schematic way, the responses of the gerbil brain to unilateral carotid artery occlusion, which led to partial ischemia. In this case, the cerebral blood flow (CBF) decreased in two stages due to the partial ischemia that developed. The decrease in CBF was concomitant and correlated to the increase in the mitochondrial NADH and a slow increase in the extracellular level of $K^{+}$. At a certain point of the partial ischemic event, ischemic depolarization (ID) was developed. As seen, the CBF decreased to its minimal level together with the increase in NADH. The ionic homeostasis was disturbed completely as seen by the steep increase in the $K^{+}$ and a rapid decrease in extracellular ${\mathrm{Ca}}^{2+}$. Moreover, a large increase in the R trace was recorded due to a massive constriction of blood vessels in the microcirculation. After the reopening of the carotid artery, all parameters returned to the preischemic levels within 5 to 10 min. To evaluate the protective effects of a tested compound, various parameters could be calculated regarding timing or amplitude values, as seen in the figure.

#### 3.3.3 From animal studies to human brain monitoring

In my laboratory, we monitored a large number of organs in different animals although the brain in rats was our main organ and animal model.

**Fig. 11:**
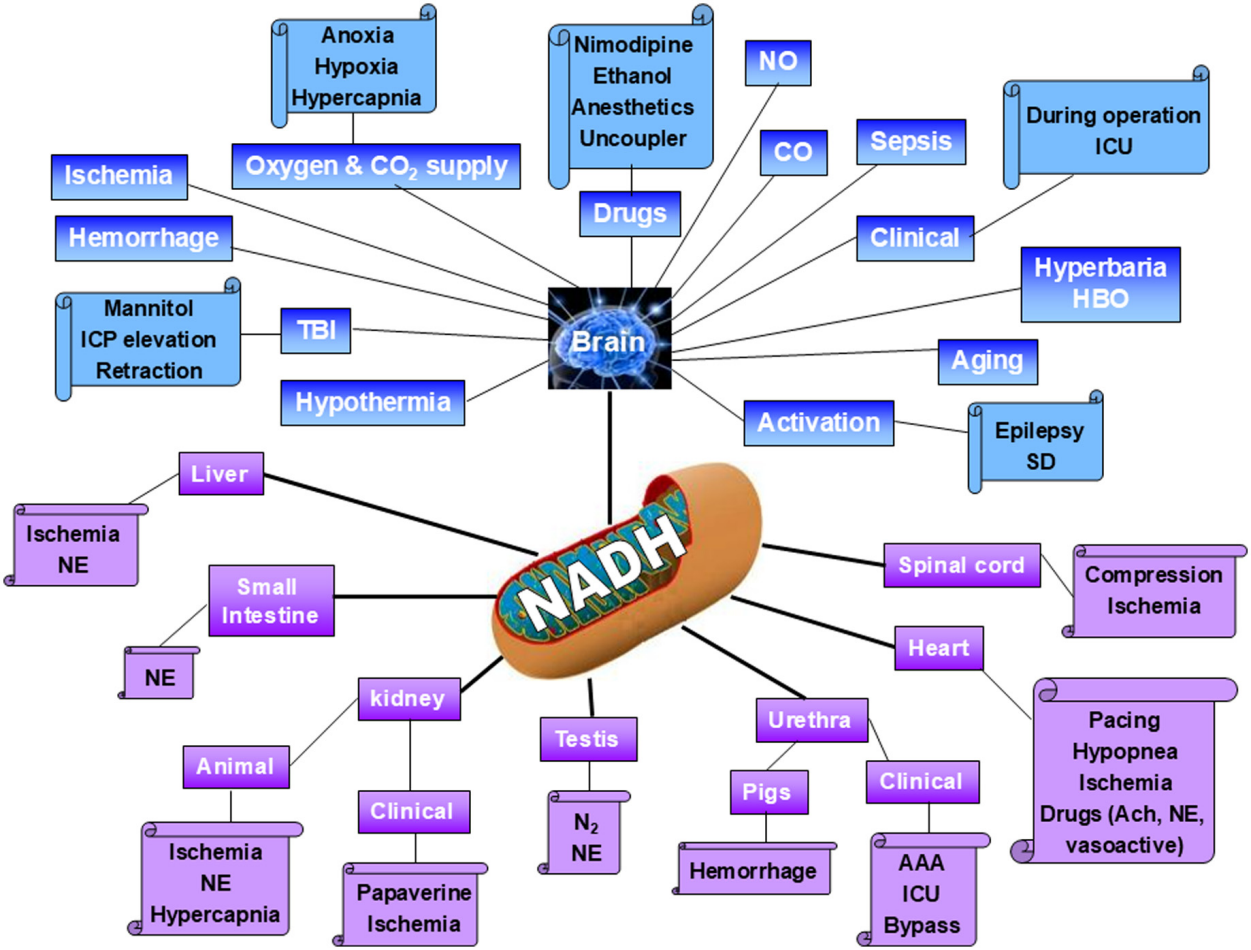
Presentation of the monitored organs and perturbations measured by Mayevsky et al. in experimental animals as well as in patients.^35^ For details, see Ref. 38.

Figure 11 summarizes the spectrum of organs monitored, by our group, under various perturbations in experimental animals as well as in patients. As could be seen, we monitored various organs, but the main organ studied in my laboratory was the brain in rats and gerbils. Table 7.1 of chapter 7 in my book^38^ lists the published papers by Mayevsky and collaborators according to various organs in general and the brain according to the perturbations used.

The new era in monitoring of mitochondrial function in patients started in 1990, when our team developed a unique multiparametric monitoring system that included the measurement of NADH fluorescence, using a light guide–based device.^55^ This system was initially applied to monitor neurosurgical patients undergoing brain surgery. In the second half of the 1990s,^48^^,^^56^^,^^57^ we were able to monitor the brain of comatose patients in the ICU and described, for the first time, the development of CSD-like responses, including marked NADH responses that were correlated to the microcirculatory blood flow. A modified multiparametric monitoring system was applied for NADH measurement during neurosurgical procedures in the operating room.^58^^–^^61^

**Fig. 12:**
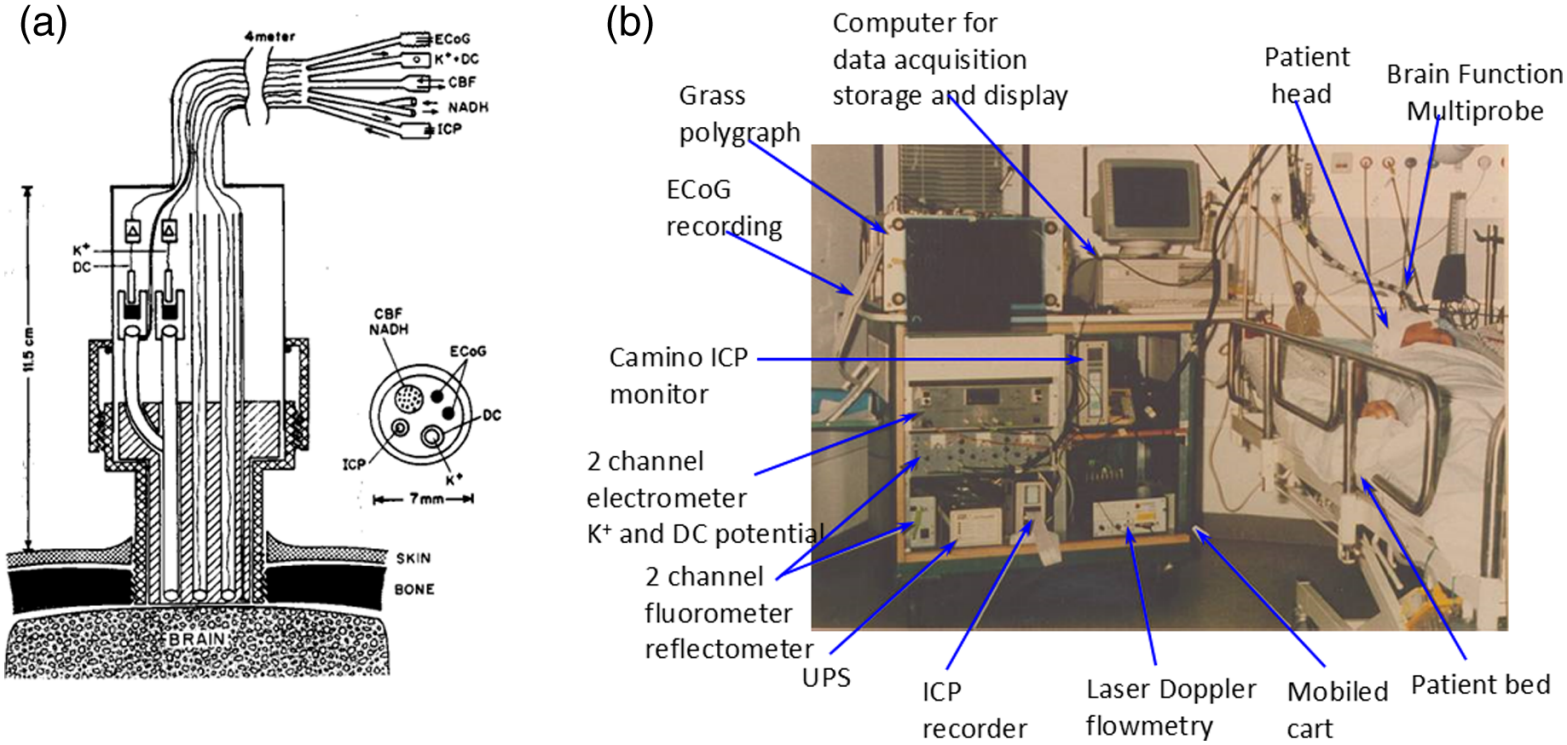
(a) Schematic representation of the MPA used for monitoring the brain of head-injured patients in the ICU. The MPA is connected to the brain via a special holder screwed into the skull. ICP, intracranial pressure probe; CBF, NADH-fiber optic light guide probe to measure local cerebral blood flow (CBF) and mitochondrial redox state (NADH); K+, DC, extracellular K+ mini surface-electrode surrounded by a DC steady potential monitoring space; ECoG, bipolar electrocortical electrodes.^48^ (b) The first clinical monitoring setup (“brain monitor”) used in the neurosurgical intensive care unit. The multiparametric monitoring system consists of various devices installed in the same cart. The MPA shown in Fig. 12(a) is connecting the brain of the patient to the brain monitor.^62^

The ideal system was to monitor eight parameters from the brain (Fig. 9) in addition to the various systemic parameters monitored in every patient hospitalized. The long-term vision was that all parameters monitored will be integrated into the same data bank, and an expert system will be developed for better diagnosis of the patients. The translation of this concept into a practical tool and monitoring device is presented in Figs. 12(a) and 12(b).

Based on the well-developed MPA used for animal experiments,^47^ a new MPA was developed and applied to patients monitored in the neurosurgical operation rooms and ICU. This device allowed us to monitor, in real time, the hemodynamic, metabolic ionic, and electrical activities in the brain of comatose patients. All details regarding the technology and the clinical setup appear in our published paper.^48^ Figure 12(a) shows the longitudinal section of the MPA (measuring eight parameters) used in the neurosurgical operating room and ICU. The MPA was connected to the multiparametric monitoring system shown in Fig. 12(b).

**Fig. 13:**
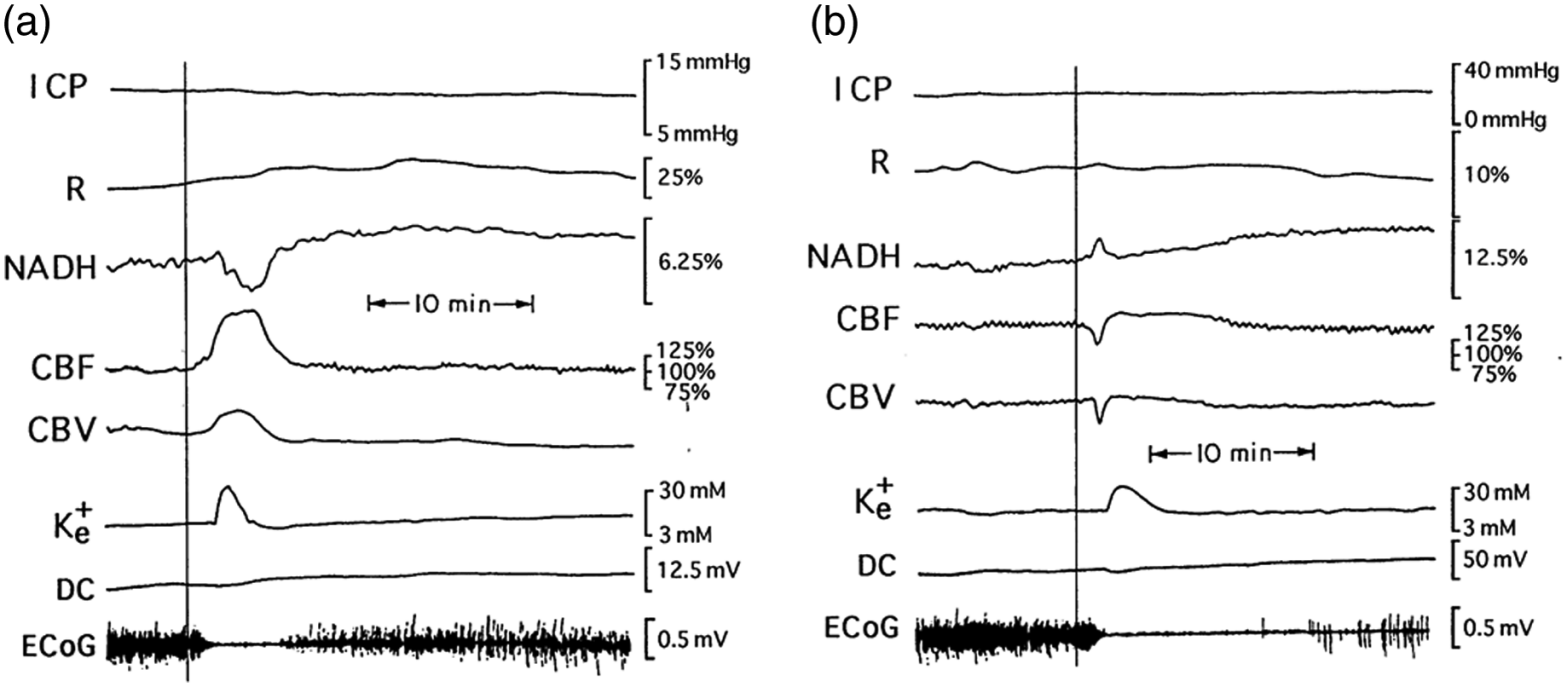
(a) Responses of the brain to wave of CSD developed spontaneously in a severely head-injured patient. ICP, intracranial pressure; R, 366-nm reflectance; NADH, 450-nm corrected fluorescence; CBF, CBV, cerebral blood flow and volume measured by laser Doppler flowmetry; ${K^{+}}_{e}$, DC, extracellular potassium levels and the DC steady potential measured around the $K^{+}$ electrode; ECoG, electrocorticography.^48^ (b) Repeated response to CSD in the same patient.^56^

The next step was to use the multiparametric monitoring system in the neurosurgical ICU. In this group, 14 patients were monitored (Fig. 13). Only one of the 14 monitored patients had developed spontaneous changes in all parameters similar to the typical responses to spreading depression (SD) in animals. These changes were recorded 4.5 h after the beginning of monitoring, which was 7 h after admittance to the hospital. During the measuring period, this patient was bilaterally irresponsive to pain; his pupils were dilated and non-reactive to light. He was mechanically ventilated, and his brain CT scan showed evidence of severe brain edema in the left hemisphere and right parietal hemorrhagic contusion. The measurements were taken from the right frontal lobe.

As seen in Fig. 13(a), the Electrocorticogram (ECoG) became depressed for 10 to 15 min, and at the same time, a cycle of elevated extracellular $K^{+}$ and a small negative shift in the direct current (DC) potential were recorded. These changes are typical of transient depolarization of the neurons and glial cells, which is a dominant part of CSD. NADH was oxidized while blood flow and volume increased. This patient exhibited repetitive SD cycles every 20 to 30 min. However, the following SD-like cycles that were recorded from this patient (after the first ones) showed different hemodynamic and metabolic responses [Fig. 13(b)]. Although the extracellular level of $K^{+}$ and the pattern of the DC potential were very similar, NADH oxidation cycles were replaced by a biphasic cycle comprised mainly of a phase of increased NADH followed by a small oxidation phase. The compensation of blood flow and volume was also reversed at this time. The monophasic increase in CBF and CBV was replaced by an initial decrease followed by a smaller increase. Significant correlations were seen among CBF, CBV and NADH (CF), and extracellular $K^{+}$ levels.

## 4 Discussion

### 4.1 Monitoring of NADH Fluorescence

The fact that NADH was monitored by the difference in the absorption spectrum of its reduced form limited the use of that technique to the study of mitochondria *in vitro* and in very thin tissue samples (e.g., muscle) or in cell suspension. To provide a method more specific than absorption spectroscopy, fluorescence spectrophotometry in the near-ultraviolet range (UV-A) was applied for NADH measurement. The initial model of fluorescence recorder was described by Theorell & Nygaard^63^^,^^64^ and Theorell et al.^65^ Using a modified device, Boyer and Theorell^66^ showed that the fluorescence of diphosphopyridine nucleotide (DPNH) was shifted, and the intensity was increased upon the combination of DPNH and liver alcohol dehydrogenase-ADH. The first detailed study using fluorescence spectrophotometry of NADH in intact Baker’s yeast cells and algae cells was published in 1957 by Duysens and Amesz.^67^

In the next 5 years (1958 to 1962), the monitoring of NADH fluorescence was significantly expanded, led by Chance and collaborators. In a first preliminary study, Chance et al.^68^ performed simultaneous fluorometric and spectrophotometric measurements of the reaction kinetics of bound pyridine nucleotides (PN) in the mitochondria. In the same year (1958), Chance and Baltscheffsky^69^ presented preliminary results of measuring the fluorescence of intramitochondrial PN. In this study, they proved the connection between the mitochondrial metabolic state and the redox state of NADH as measured by spectral fluorometry in mitochondria isolated from rat liver.^70^

The correlation between the enzymatic assay of PN and sensitive spectrophotometry was investigated by Klingenberg et al. using rat liver, heart, kidney, and brain.^71^

In 1959, Chance and collaborators were able to expand the use of NADH fluorometry to various experimental models, from isolated mitochondria to intact tissue. Intramitochondrial pyridine nucleotides were analyzed in connection to the ADP-ATP cycle.^72^ To monitor NADH localization in intact cells, Chance and Legallias^73^ developed a unique differential microfluorometer with a very high spatial resolution. This approach was used, in various cells, to identify the intracellular localization of NADH fluorescence signals.^74^^–^^76^ The next step was to apply the fluorometric technique to the higher organization level of animal tissues. Together with Jobsis, Chance measured *in vitro* changes in muscle NADH fluorescence following stimulation.^77^ In another paper, Chance and Thorell^78^ came to the very significant conclusion that “The oxidation and reduction state of mitochondrial pyridine nucleotide without a measurable change of cytoplasmic fluorescence suggest that compartmentalization of mitochondrial and cytoplasmic pyridine nucleotide occurs *in vivo*, at least in the grasshopper spermatid.” In another paper, Chance and Hollunger^79^ elaborated on the energy-linked reduction of the mitochondrial pyridine nucleotides.

An intensive use of the *in vivo* NADH monitoring approach started in 1962. The “classical” paper on *in vivo* monitoring of NADH was published in 1962 by Chance et al.^1^ They were able to simultaneously monitor the brain and kidney of anesthetized rats using two microfluorometers. In 1962, Chance and collaborators elaborated on this kind of *in vivo* monitoring and used it in other rat organs.^80^^–^^82^

The main limitation of the technology used in monitoring tissues’ NADH fluorescence was the optical system that transmits the excitation as well as the fluorescence light to the tissue and to the detection system. The availability of optical fibers that transmit light in the UV part of the spectrum in the early 1970s opened up new options to monitor tissue NADH fluorescence and reflectance from the surface of the brain as well as other organs *in vivo*.

**Fig. 14:**
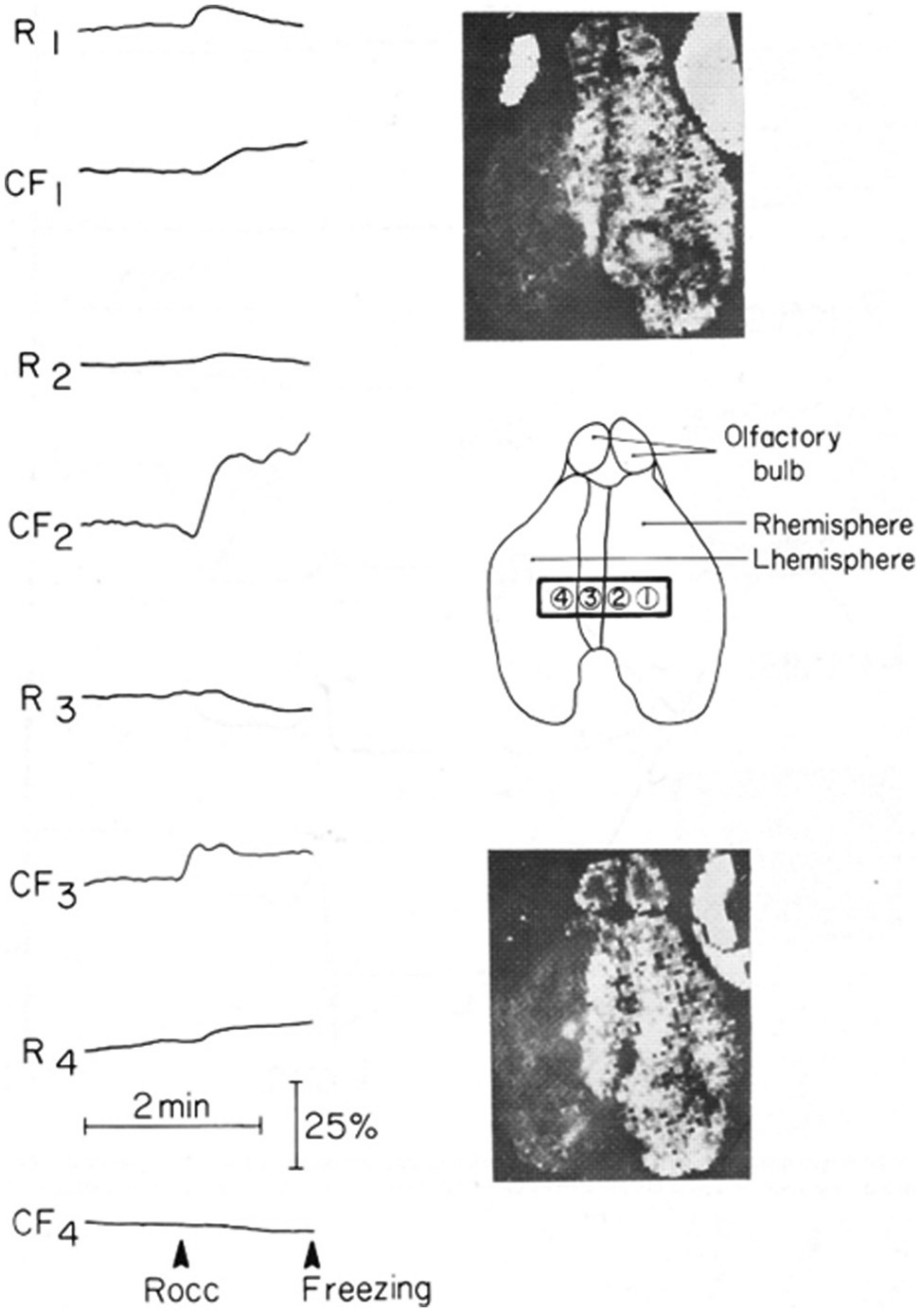
Effects of right carotid occlusion on NADH fluorescence measured *in vivo* and in the frozen brain of a normal (Tumblebrook Farm) gerbil. Site 3 located above the midline area of the left hemisphere did respond to the contralateral carotid occlusion. The two scans shown are of the PN and represent different depths in the brain.^83^

A very important advantage of fiber optic–based fluorometry was found in studying the correlation between the anatomy of the blood vessels to the brain and the metabolic responses of the mitochondria monitored *in vivo*.^83^ To clarify the interhemispheric transfer paradox, we used a four-channel light guide (1-mm diameter for each common part) held in a special micromanipulator and placed above the two hemispheres, as shown in the schematic part of Fig. 14. Unilateral carotid occlusion (left or right) and bilateral occlusion were used, and the NADH responses *in vivo* were recorded. In gerbils that exhibited the interhemispheric transfer, the results were similar to those presented in Fig. 14 (left side), namely, during unilateral occlusion, three out of four areas were affected and NADH levels were higher in comparison to the fourth site. In a few gerbils, one area was affected, and the other three were not affected by the unilateral occlusion. To obtain the frozen brain data, unilateral occlusion was done, the light guides were removed quickly, and the brain was frozen by the funnel technique described previously. The 2D distribution of the PN redox state in two slices of the brain is shown below and above the scheme in Fig. 14.

## 5 Conclusions and Future Outlook

It is now almost 70 years since the publication of Chance and Williams (1955) summarizing various aspects of mitochondrial metabolic state *in vitro*. A few years later, in the early 1960s, the monitoring of mitochondrial NADH *in vivo* was established in Philadelphia,^1^ and many groups of scientists around the world adopted the approach and published 800 to 900 papers as presented and discussed in my book.^38^ My collaboration with the giant scientist, Professor Britton Chance, started in 1972 when I arrived in Philadelphia as a young post-doc fellow. The last time we met was during our stay in China at the Britton Chance Center for Biomedical Photonics established at the WNLO, HUST Wuhan, China. During our many years of collaboration, he always mentioned the need to translate the technology and data collected in experimental animal studies into a practical clinical tool to help mankind. In chapter 9 of my book,^38^ I summarized all the published papers regarding the monitoring of NADH redox state in patients.

In the last years, we were able to develop a practical tool that was approved by the FDA and was tested in preliminary clinical studies.^84^ As discussed in chapter 9 of my book, the concept of clinical monitoring of mitochondrial NADH as well as microcirculatory blood flow and oxygenation is well accepted. I have good reasons to believe that the multiparametric monitoring approach will be the standard tool in daily patient monitoring.

The next step in a patient monitoring device will be a very easy-to-use and practical tool as well as the interpretation of the monitored parameters into a new tissue vitality index that was discussed in my book.

Calibration of NADH fluorescence as well as the other monitored parameters in absolute units will be the next goal of the team dealing with patient monitoring.

## Data Availability

The submitted paper describes the basic scientific information regarding the concept of monitoring mitochondrial NADH redox state using UV-transmitting optical fibers to illuminate animal tissues under *in vivo* conditions. Moreover, the emitted reflected light from the tissue is transmitted by another group of optical fibers. The basic information on this technology was summarized in a book published by Mayevsky^38^ (mainly in chapter 5).
